# Granulosa-Lutein Cell Sirtuin Gene Expression Profiles Differ between Normal Donors and Infertile Women

**DOI:** 10.3390/ijms21010295

**Published:** 2019-12-31

**Authors:** Rebeca González-Fernández, Rita Martín-Ramírez, Deborah Rotoli, Jairo Hernández, Frederick Naftolin, Pablo Martín-Vasallo, Angela Palumbo, Julio Ávila

**Affiliations:** 1 Laboratorio de Biología del Desarrollo, UD de Bioquímica y Biología Molecular and Centro de Investigaciones Biomédicas de Canarias (CIBICAN), Universidad de La Laguna, Av. Astrofísico Sánchez s/n, 38206 La Laguna, Tenerife, Spain; refernan@ull.edu.es (R.G.-F.); rmartira@ull.edu.es (R.M.-R.); deborah_rotoli@yahoo.it (D.R.); pmartin@ull.edu.es (P.M.-V.); 2 Institute of Endocrinology and Experimental Oncology (IEOS), CNR-National Research Council, 80131 Naples, Italy; 3 Centro de Asistencia a la Reproducción Humana de Canarias, 38202 La Laguna, Tenerife, Spain; jairoh@fivap.com (J.H.); apalumbo@fivap.com (A.P.); 4 Department of Obstetrics and Gynecology, New York University, New York, NY 10016, USA; frederick.naftolin@nyulangone.org

**Keywords:** granulosa-lutein cells, sirtuin, PCOS, poor responders, endometriosis, infertility diagnosis

## Abstract

Sirtuins are a family of deacetylases that modify structural proteins, metabolic enzymes, and histones to change cellular protein localization and function. In mammals, there are seven sirtuins involved in processes like oxidative stress or metabolic homeostasis associated with aging, degeneration or cancer. We studied gene expression of sirtuins by qRT-PCR in human mural granulosa-lutein cells (hGL) from IVF patients in different infertility diagnostic groups and in oocyte donors (OD; control group). Study 1: sirtuins genes’ expression levels and correlations with age and IVF parameters in women with no ovarian factor. We found significantly higher expression levels of *SIRT1*, *SIRT2* and *SIRT5* in patients ≥40 years old than in OD and in women between 27 and 39 years old with tubal or male factor, and no ovarian factor (NOF). Only *SIRT2*, *SIRT5* and *SIRT7* expression correlated with age. Study 2: sirtuin genes’ expression in women poor responders (PR), endometriosis (EM) and polycystic ovarian syndrome. Compared to NOF controls, we found higher *SIRT2* gene expression in all diagnostic groups while *SIRT3*, *SIRT5*, *SIRT6* and *SIRT7* expression were higher only in PR. Related to clinical parameters *SIRT1*, *SIRT6* and *SIRT7* correlate positively with FSH and LH doses administered in EM patients. The number of mature oocytes retrieved in PR is positively correlated with the expression levels of *SIRT3*, *SIRT4* and *SIRT5*. These data suggest that cellular physiopathology in PR’s follicle may be associated with cumulative DNA damage, indicating that further studies are necessary.

## 1. Introduction

Sirtuins (silent information regulator proteins) were initially defined as an evolutionarily conserved family of class III nicotinamide adenine dinucleotide+ (NAD+)-dependent histone deacetylases that can also have mono-ADP-ribosyltransferase activity [1,2]. These enzymes cleave an acetyl group from acetyl-lysine residues in histones, although they can also act in nonhistone proteins, such us structural proteins, metabolic enzymes or transcriptional factors [1,2]. However, recent studies demonstrate that sirtuin family gene products catalyze additional reactions and enzymatic activities, including mono-ADP-ribosyltransferase, deacylase, deacetylase, desuccinylase, demalonylase, demyristoylase and depalmitoylase activities [2,3,4]. 

Sirtuins are implicated in aging, oxidative stress, maintenance of metabolic homeostasis, DNA repair and mitochondrial function [5,6] through the regulation of specific genes’ expression and activation or deactivation of other proteins [7,8]. 

In mammals, seven sirtuin isoforms have so far been described (*SIRT1*–*SIRT7*). These possess a conserved catalytic domain but differ in their amino and carboxyl terminal that confers specificity in cellular location and function [9]. 

*SIRT1* is the most studied member of the sirtuin family, it is located mainly in the nucleus, although it shuttles to the cytosol in response to environmental signals [10]. *SIRT1* has been implicated in processes such as inflammation, by reducing NF-κB activity [11,12], apoptosis by inhibiting p53-dependent transcription [13,14] and in energy metabolism through effects on regulators of metabolic enzymes such as PPAR-γ [15]. Yeast Sir2 gene is a *SIRT1* homolog that is involved in yeast life span extension, nonetheless, but a similar role for *SIRT1* has been refuted [16]. In the reproductive system, *SIRT1* plays a role in apoptosis of granulosa cells during follicular atresia [17,18] and has been related to preservation of follicular reserve and extension of ovarian lifespan [19].

*SIRT2* is located in the cytoplasm [9]. It transiently migrates into the nucleus to deacetylate α-tubulin and to modulate chromatin condensation and cell cycle regulation by deacetylating H3 and H4 histones [20,21]. *SIRT2* deacetylates transcriptional factors like Foxo, p53 or NF-κB [4,22,23,24].

*SIRT3* resides in the mitochondria [9] and participates in the regulation of energy metabolism [25,26] and apoptosis [27] and in reactive oxygen species (ROS) detoxification [28,29]. *SIRT3* has been involved in age-associated oxidative stress and infertility [30,31,32].

*SIRT4* located in the mitochondria [9] regulates lipid metabolism promoting fatty acid oxidation and inhibiting lipogenesis [33,34]. The effects of *SIRT3* and *SIRT4* on glutamate dehydrogenase are opposite: while *SIRT4* represses GDH activity, deacetylation by *SIRT3* activates GDH [35,36].

*SIRT5* in mitochondria [9,37] has been reported to possess low deacetylase activity compared to the other members of the family. In contrast, *SIRT5* possesses high desuccinylation, demalonylation and deglutarylation activities [4]. It activates the urea cycle by deacetylating carbamoyl phosphate synthetase 1 [38]. *SIRT5* desuccinylates isocitrate dehydrogenase 2 and deglutarylates glucose-6-phosphate dehydrogenase, which protect cells from oxidative damage by activating NADPH-producing enzymes [39]. In women, *SIRT5* expression decreases along with ovarian reserve as maternal age increases [40].

*SIRT6*, nuclear [10], is implicated in telomeres stabilization, DNA double strand break repair and regulation of transcription [41,42,43,44]. *SIRT6* overexpression increases lifespan in male mice by ~15% [45]. Regarding fertility, *SIRT6* has been associated with follicle reserve preservation and increase of ovarian function lifespan [19].

*SIRT7*, predominantly localized in the nucleolus [9], co-activates ribosomal DNA transcription by association with RNA polymerase I complex [46,47]. In addition, *SIRT7* interacts with chromatin remodeling complexes by association and deacetylation of the B-WICH component [48] and plays a role in stress resistance to hypoxia, osmotic stress, ER-stress or genomic stress [49,50,51]. 

The aim of this study was to investigate the expression of the genes coding for the 7 sirtuins in human granulosa–lutein (hGL) cells from in vitro fertilization (IVF) patients with different infertility diagnoses, aging women and oocyte donors (young controls) in order to seek differences in gene expression levels and possible correlation with clinical parameters.

## 2. Results

### 2.1. Descriptive Statistics and Clinical Variables 

Significant differences in age distribution were found between OD (oocyte donors between 18 and 27 yo) and all other groups (*p* = 0.000) and between ≥40 yo (women ≥ 40 yo with tubal or male factor and no ovarian factor) and all other groups (*p* = 0.000). No age difference was observed between NOF (women between 27 and 39 yo with tubal or male factor and no ovarian factor), PCOS (polycystic ovarian syndrome), EM (endometriosis) and PR (women < 40 yo defined as poor responders) (Table 1).

While agonist and antagonist protocols were differently represented between diagnostic groups, within each group there were no statistically significant differences in gene expression between the 2 protocols. 

Regarding the amount of exogenous gonadotropins used for ovulation induction, PCOS, OD and NOF groups received significantly lower doses compared to EM, PR and ≥40 yo (*p* = 0.000). The number of total and mature oocytes retrieved varied between groups: significant differences are shown in Table 1. No statistically significant differences in mean E2 peak value were observed among groups.

### 2.2. Sirtuin Gene Expression 

#### 2.2.1. Study 1: Sirtuin Gene Expression Level and Correlations with Age and IVF Parameters in Women with No Ovarian Factor

All sirtuin family members were expressed in human granulosa-lutein cells, Table 2. *SIRT1* and *SIRT2* were the most expressed. *SIRT3*, *SIRT5* and *SIRT7* showed an intermediate expression and *SIRT4* and *SIRT6* the least expression.

**Figure 1:**
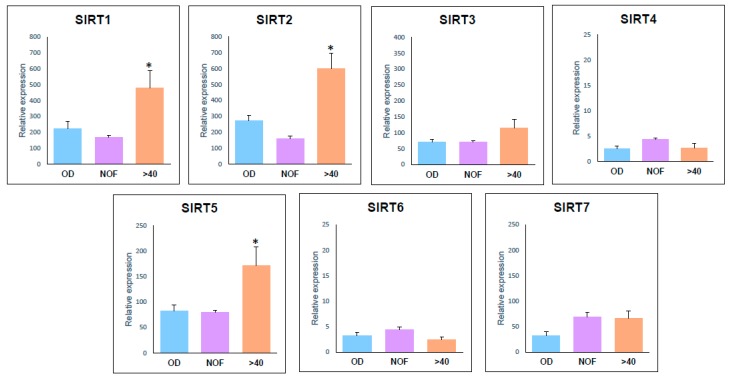
Histogram representation of expression levels of sirtuin genes in OD, NOF and >40 groups. Statistically significant different means (optical densities) compared to the control group are marked by an asterisk (*).

Comparing expression between different IVF diagnostic groups gene expression was higher in ≥40 yo patients than in other groups for *SIRT1* (OD: *p* = 0.008; NOF: *p* = 0.000), *SIRT2* (OD: *p* = 0.000; NOF: *p* = 0.000) and *SIRT5* (OD: *p* = 0.032; NOF: *p* = 0.014). With the exception of *SIRT2* and *SIRT7* the NOF group did not differ from the controls, Figure 1.

**Figure 2:**
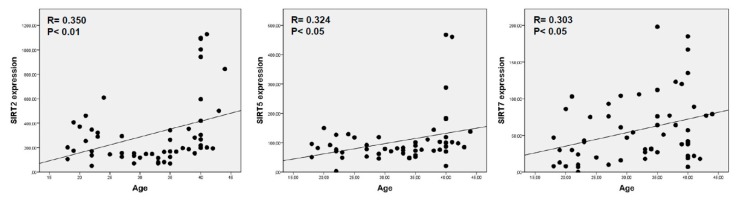
Graphic representation of correlation between *SIRT2*, *SIRT5* and *SIRT7* and age.

Sirtuins and age—When all women with no ovarian factor (OD, NOF and ≥40 yo) were analyzed, we observed that only *SIRT2*, *SIRT5* and *SIRT7* expression correlated with age (r = 0.350, *p* < 0.01; r = 0.324, *p* < 0.05 and r = 303, *p* < 0.05) (Figure 2). 

Sirtuins and IVF parameters—When different groups were analyzed, no correlation was found between NOF or ≥40 yo with any IVF parameters. The ovum donor controls showed a negative correlation between *SIRT2*, *SIRT3*, *SIRT4* and *SIRT6* and administered gonadotrophin doses (*p* < 0.01) and a negative correlation between *SIRT7* and FSH doses (*p* < 0.05). In addition, *SIRT2*, *SIRT4*, *SIRT6*, *SIRT7* expression also correlated negatively with total treatment days (*p* < 0.05). 

#### 2.2.2. Study 2: Correlations between Sirtuin Gene Expression and IVF Parameters in Women with Different Infertility Diagnosis compared to NOF.

**Figure 3:**
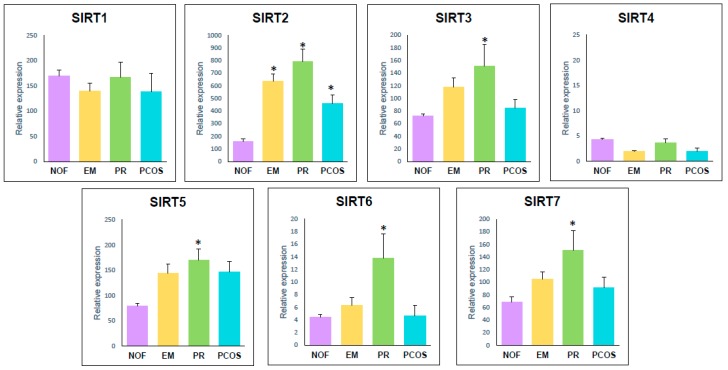
Histogram representation of expression levels of sirtuin-1–7 gene expression in NOF, EM, PR and PCOS groups. Statistically significant different means with respect to the NOF group are marked by an asterisk (*).

Comparing expression between different groups (Table 2), no significant difference was observed in *SIRT1* and *SIRT4* gene expression level among any group. Expression levels of *SIRT2* were statistically higher in EM (*p* = 0.000), PR (*p* = 0.000) and PCOS (*p* = 0.010) patients compared to NOF. However, PR present the higher expression level, statistically different from PCOS (*p* = 0.008), while EM has an intermediary value that did not differ from any other group, Figure 3. *SIRT3*, *SIRT5* and *SIRT7* gene expression was statistically higher in PR than in NOF (*p* = 0.029; *p* = 0.011; *p* = 0.003). PCOS and EM present an intermedia expression value that did not differ from NOF or PR (Figure 3). *SIRT6* expression was statistically higher in PR patients than in all other groups (NOF: *p* = 0.002; EM: *p* = 0.045; PCOS: *p* = 0.008) (Figure 3). 

When different diagnostic groups were analyzed, we observed that in PR, ≥40 yo and PCOS groups no correlation was found between sirtuin gene expression and gonadotrophin doses. Only in EM patients did *SIRT1*, *SIRT6* and *SIRT7* correlate positively with FSH and LH doses administered. Interestingly, these correlations with *SIRT6* and *SIRT7* were opposite to those found in control OD. 

With the exception of a negative correlation for *SIRT4* in PCOS patients, no correlations were found between sirtuin expression and number of days of treatment in the analyzed groups.

There was a positive correlation between mitochondrial sirtuins and mature oocytes retrieved in PR patients.

## 3. Discussion

### 3.1. These Are Novel Findings Regarding the Possible Role of the Sirtuin Gene Family in Reproductive Failure

In view of their NAD+ dependence [51], there may be a role for sirtuins in intrafollicular cell redox balance and antioxidant response.

This article reports the expression levels of the seven sirtuin genes presently known in post-ovarian stimulation and human chorionic gonadotrophin treated human hGL cells and establishes correlations among sirtuin gene expression and clinical status: age, clinical diagnosis, specific ovulation induction and response to treatment; Table 1. Notably, the average estradiol level in the PR group is relatively elevated. We defined PR based on the Bologna criteria [52], but excluding the advanced maternal age subjects, which represent a separate group in our study. The stimulation protocol was adjusted to furnish satisfactory serum estradiol levels prior to hCG injection. We used an antagonist protocol with high doses of LH and FSH based on the expected poor response and the high estradiol levels obtained in some patients probably reflect the high dose of LH used for ovulation induction. Interestingly, our group of poor responders does not include patients >40; the patients’ age range is 28–39, which may have positively affected the ability of granulosa cells to produce estrogen. Our results are in agreement with the paper by Ku et al. [53], which showed higher estradiol levels in patients treated with a combination of FSH and LH. However, Ku el al. used an ultralong protocol, which may be responsible for lower estradiol levels in their patients.

IVF patients with no ovarian factor (OD, NOF and ≥40 yo) showed a direct correlation between their hGL cell sirtuin 2, 5 and 7 gene expression and age. This could be a means of maintenance of energy homeostasis, glucose metabolism and reactive oxygen species detoxification, DNA repair and other cell repair mechanism that decrease with age and could result is poor response to ovarian stimulation and compromised oocytes [54,55].

The positive correlation of *SIRT2* expression levels with age (*p* < 0.01) in women with no ovarian factor shows a pronounced increase at 40 years old, as shown in Figure 2 scatter plot. The expression levels of *SIRT2* in the PR and EM groups of patients resemble those of ≥40 yo women, Table 2. The higher expression levels of *SIRT2* gene in women older than 40 yo than in the OD and NOF groups supports the hypothesis that *SIRT2* expression increases most notably after forty years [56,57,58], always within the age limits or our study.

*Summary*—Taken together, these findings imply a feedback loop that induces sirtuin 2, 5 and 7 expression with (ovarian) aging and in relation to disease states that foster the presence of reactive oxygen species in or around the ovary. However, these descriptive studies were not designed to test such relationships.

### 3.2. These Findings Have Implications for Ovarian Aging.

Fertility decreases from age 35 and reaches a clinically perilous point in most women by age 40. In our study, *SIRT1* gene expression levels were higher in women older than 40 yo than in OD and NOF patients (Figure 1). These data are in agreement with those of Di Emidio et al. [30], who reported higher levels of *SIRT1* mRNA in aged mouse oocytes and a decreased ability to react to H_2_O_2_ addition as a sign of increased oxidative stress (OS). This coincides well with the increase of *SIRT1* expression in response to age dependent OS. A direct correlation between aging and OS increase has been reported with aging-related decay of fertility [59], and our laboratory reported an increased expression levels of the oxidative stress response gene ALDH3A2 in granulosa-lutein cells that is related to age and infertility diagnosis [60,61].

*SIRT5* gene expression level is significantly higher in ≥40 yo than in OD and NOF (*p* = 0.045 and *p* = 0.010, respectively). *SIRT5*, localized in the mitochondrial matrix, regulates mitochondrial activity and function [62] by succinylating [63] or acetylating proteins such as cytochrome C, which is pivotal in oxidative metabolism and the apoptosis processes [36]. 

These studies closely link sirtuins to the aging ovary. The increase in expression of *SIRT5* gene in women older than 40 years old could represent a feedback loop that is related to the presence of oxidative species.

*Other studies of the sirtuin family and the ovary*—There have been other studies of *SIRT5* in human granulosa-lutein cells. Pacella-Ince et al. [40] reported a lower gene expression level in women of advanced maternal age and reduced ovarian reserve compared to younger women, which is apparently opposite to our results. However, in the article by Pacella-Ince et al., the patients’ distributions within each age group include no ovarian factor women as well as different infertility diagnoses. Furthermore, the different infertility diagnoses are not represented in all groups and percentages among them are notably variable; as a result, the amount of *SIRT5* gene expression given is not specific for age, but just an average of the combined groups [40].

While convincing of differences in mural hGL cells, these findings do not reveal sirtuin family expression in the cumulus cells that are directly in apposition to the oocyte/embryo. We have shown that there are important biochemical differences between mural and cumulus cells [64,65]. Additionally, there have been reports of miRNA differences in poor responders [66] and other genes’ expression in cumulus cells from obese women [67,68]; the possibility of mural vs. cumulus differences is on the list for further study.

*Summary—*The human ovarian follicle, represented by post-ovulatory luteinized granulosa cells, expresses all known members of the sirtuin family of genes. We have proposed a feedback mechanism related to the presence of inflammation/reactive oxygen species/aging that drives expression of sirtuins as a defensive/protective mechanism. *SIRT6* and *SIRT7* functions include regulation of DNA-repair mechanisms [43,44,50,69]. The overexpression of *SIRT6* and *SIRT7* in the PR group of patients might be indicative of the role of these sirtuins in an attempt to repair accumulated damage to DNA caused by a deficient response to cellular genotoxic stress [45,69]. However, in NOF and ≥40 yo, the expression levels of these sirtuins do not differ from those observed in younger women (OD) (Table 2). This suggests that in PR the DNA damage is mainly due to genomic instability and not affected by aging [54]. In the case of patients suffering from EM or PCOS, the slight increase expression of *SIRT7* gene observed may be an effect of incorrect balance between redox response mechanisms and the higher oxidative stress described for these pathologies.

**Figure 4:**
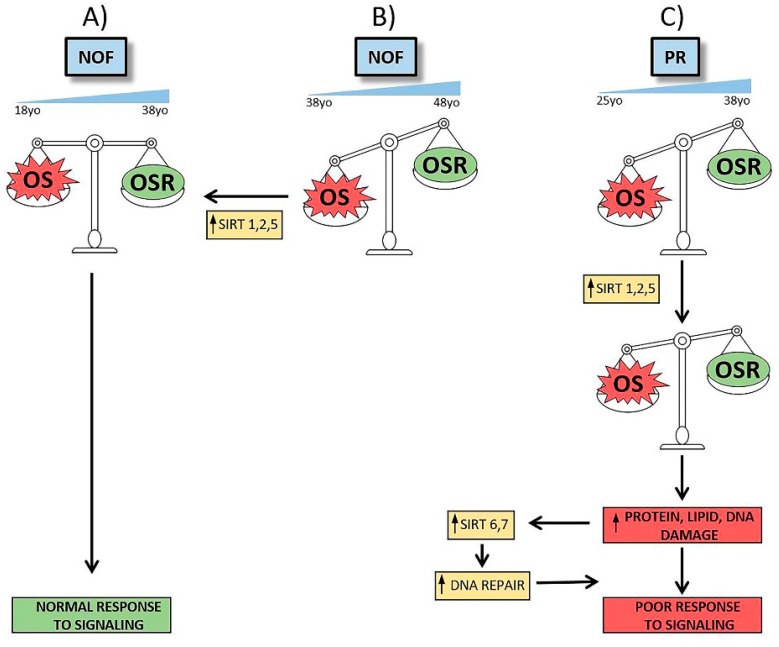
Scheme of alterations in NOF and PR groups and possible sirtuins’ roles. According to our studies, NOF women between 18 to 38 years old may avoid OS damage by OSR, triggering a normal signaling response and maintaining an equilibrated OS/OSR status (**A**). It is possible that this equilibrium favors OS during pre-menopausal aging (**B**). In that case, OSR are not sufficient to protect cells from OS actions and *SIRT1*, *SIRT2* and *SIRT5* gene expression increase would be required to recover homeostasis. Failing this response, women poor responders show a different sirtuin pattern (**C**). In this group, women between 25 to 38 years old have an imbalance between OS and OSR similar to older NOF women. Cellular attempts to reach homeostasis by increasing *SIRT1*, *SIRT2* and *SIRT5* gene expression are insufficient and it is necessary activate protein, lipid and DNA repair mechanisms and others sirtuins’ expression. Despite the fact that *SIRT6* and *SIRT7* gene expression increase, cells cannot response to signaling and homeostasis cannot be recovered, leading to a clinically poor response to follicle stimulation.

The diverse expression patterns of sirtuins among groups reveals not only a clinical, diagnosis-specific profile, but also an age dependent feature. Figure 4 presents a scheme of alterations and proposed compensatory responses by sirtuin expression in aging and in environmental and metabolic circumstances reflecting pathological and physiological conditions.

## 4. Materials and Methods 

### 4.1. Patients

Under a protocol approved by the Ethics Committee of the Universidad de La Laguna, women undergoing ovulation induction for oocyte donation or IVF consented to join this study. Upon reaching the target follicle sizes and receiving 250 µg of hCG, the harvesting of oocytes was by standard methods, see below. After removal of the oocyte from the petri dish, the accompanying mural granulosa-lutein cells were set aside for evaluation, see below. Clinical information included the doses of exogenous follicle stimulating hormone (FSH) and luteinizing hormone (LH) administered, and IVF parameters related to ovarian response to ovulation induction (number of total and mature oocytes retrieved, estradiol concentration on the last day of stimulation, and total number of days of stimulation). 

#### 4.1.1. Study 1: Women with No Ovarian Factor 

Fifty-six women between 18 and 44 years of age (yo) with no ovarian factor were grouped as: women between 27 and 39 yo with tubal or male factor and no ovarian factor (NOF; *n* = 24); women ≥40 yo with tubal or male factor and no ovarian factor (≥40 yo; *n* = 15); and oocyte donors between 18 and 27 yo (OD, *n* = 17). 

#### 4.1.2. Study 2: Women with Different Infertility Diagnosis Compared to NOF 

Seventy-four patients were grouped as being between 27 and 39 yo with tubal or male factor and no ovarian factor (NOF; *n* = 24); women <40 yo defined as poor responders according to the European Society of Human Reproduction and Embryology (ESHRE) criteria [52] (PRs; *n* = 16); women with American Society for Reproductive Medicine (ASRM) [70] stages III and IV endometriosis, with histologic diagnosis (EM; *n* = 18); polycystic ovarian syndrome (PCOS; *n* = 16) was defined according to the Rotterdam criteria [71]. Patient demographics and the most relevant clinical characteristics of these sub-groups are shown in Table 1.

### 4.2. Ovulation Induction and Intracytoplasmic Sperm Injection (ICSI)

Ovarian stimulation was carried out with an agonist or antagonist protocol using recombinant FSH (Gonal F, Serono, Madrid, Spain), combined with recombinant LH (Luveris, Serono, Madrid, Spain) or human menopausal gonadotropins (hMG,Lepori; Farma-Lepori, Madrid, Spain or Menopur, Ferring, Madrid, Spain). Initial doses were chosen based on patients’ age and infertility diagnosis. Ovulation induction was monitored by serial ultrasounds and serum estradiol and progesterone levels. Doses were adjusted to the individual patient’s response. Ultrasound-guided egg retrieval was performed 36 h after administration of 250 µg of recombinant human chorionic gonadotropin (hCG; Serono, Madrid, Spain) or 10,000 IU of urinary hCG (Farma-Lepori, Madrid, Spain). In all cases, the fertilization method for the mature oocytes retrieved was intracytoplasmic sperm injection. Embryo transfer was carried out with a Wallace catheter under ultrasound guidance. All retrievals were performed by the same experienced operator. 

### 4.3. Isolation of hGL Cells

Mural hGL cells were collected from follicular fluid (FF) obtained during ultrasound-guided transvaginal oocyte retrieval. After removal of the oocyte, FFs from each patient were pooled, and the hGL cells lightly centrifuged. Cells were then washed in “isolation medium” (Medium 199 (Sigma-Aldrich, Missouri, MI, USA), supplemented with sodium bicarbonate (3.7 g/L) (Sigma-Aldrich), penicillin (59 mg/L) (Sigma-Aldrich), streptomycin (100 mg/L) (Sigma-Aldrich), amphotericin B (25 mg/L) (Sigma-Aldrich), L-glutamine (0.29 g/L) (Sigma-Aldrich), and bovine serum albumin (0.1%) (Sigma-Aldrich) and separated from red blood cells using a 50% Percoll (Sigma-Aldrich) gradient. Leukocytes were removed using anti-CD45-coated magnetic beads (Dynabeads M-450 CD45; Dynal ASA, Oslo, Norway) and cellular viability was confirmed by trypan blue exclusion. In all cases, it was greater than 95%.

### 4.4. Extraction of RNA

Total RNA from individual patients was extracted using Aurum total RNA mini kit (Bio-Rad Laboratories, California, CA, USA) following the manufacturer’s instructions.

### 4.5. Synthesis of Complementary DNA

RNA was reverse transcribed using “iScript cDNA Synthesis kit” (Bio-Rad Laboratories) following the manufacturer’s instructions. Total RNA was reverse transcribed in 20 µL as follows: 25 °C for 5 min and 42 °C for 30 min. The reverse transcriptase was inactivated by heating at 85 °C for 5 min. 

### 4.6. Quantitative Reverse Transcription Polymerase Chain Reaction

Quantitative reverse transcription polymerase chain reaction of complementary DNA (PCR) was employed to study the relative expression of sirtuin genes in hGL cells. All PCR was carried out using a BioRad CFX96 real-time PCR system (Bio-Rad Laboratories). The specific primers used for each sirtuin gene and the housekeeping β-actin gene used as a reference for mRNA quantification are listed in Table 3. The amplification reactions were performed in a 10 µL final volume containing 2× SsoFast EvaGreen Supermix (100 mmol/L KCl, 40 mmol/L Tris- HCl pH 8.4, 0.4 mmol/L of each nucleoside triphosphate, iTaq DNA polymerase 50 U/mL, 6 mmol/L MgCl2, SYBR Green I, 20 nmol/L fluorescein, and stabilizers (Bio-Rad Laboratories) and 0.4 µmol/L of each primer.

Each sample was analyzed in triplicate, and multiple water blanks were included in the analysis. The thermal profile used for the analysis was as follows: after a 3-min denaturation at 95 °C, 40 cycles of PCR were performed at 95 °C for 5 s and 59 °C for 5 s. Finally, a melting curve program at 65 °C to 95 °C was carried out with a heating rate of 0.1 °C/s and read every 0.5 °C. Expression levels of the genes studied are presented as individual data points as 2^−ΔCT^ [72]. Gene expression values are expressed as x105 relative to β-actin expression.

### 4.7. Statistical Analysis

Statistical analysis was performed with SPSS 23 software (IBM, New York, NY, USA). Descriptive statistics (mean and standard error (SE) are reported. One-way ANOVA followed by Tukey and Bonferroni post hoc tests were used to carry out comparisons between diagnostic groups. A Spearman rank correlation coefficient was used to assess the relationship between continuous variables. An experiment-wise α of 0.05 was chosen.

## Figures and Tables

**Table 1:** Clinical and IVF cycle parameters are shown per group.

Parameters	OD	≥40 yo	NOF	EM	PR	PCOS
N of patients	17	15	24	18	16	16
Age	22 ± 1 ^a^	41 ± 1 ^b^	34 ± 1 ^c^	36 ± 1 ^c^	36 ± 1 ^c^	33 ± 1 ^c^
Days	11 ± 1 ^a^	11 ± 1 ^a^	11 ± 1 ^a^	11 ± 1 ^a^	11 ± 1 ^a^	10 ± 1 ^a^
rFSH (IU)	2812 ± 255 ^a^	5750 ± 560 ^b^	3199 ± 391 ^a^	5622 ± 442 ^b^	6154 ± 397 ^b^	1723 ± 143 ^a^
rLH (IU)	1081 ± 161 ^a^	2645 ± 335 ^b^	1115 ± 13 ^a^	2606 ± 263 ^b^	2981 ± 274 ^b^	430 ± 70 ^a^
Peak E2 (pg/mL)	3171 ± 307 ^a^	2797 ± 188 ^a^	3054 ± 230 ^a^	2763 ± 298 ^a^	2032 ± 214 ^a^	2901 ± 321 ^a^
Total oocytes	25 ± 2 ^a^	13 ± 2 ^c/d^	17 ± 2 ^b/c^	9 ± 1 ^c/d^	6 ± 1 ^d^	21 ± 2 ^a/b^
Mature oocytes	20 ± 2 ^a^	11 ± 2 ^c/d^	13 ± 1 ^b/c^	8 ± 1 ^c/d^	5 ± 1 ^d^	18 ± 2 ^a/b^

Results are expressed as mean ± standard error. Different lowercase letters (a, b, c and d) represent statistically significant different means. OD (oocyte donors between 18 and 27 yo), ≥40 yo (women ≥40 yo with tubal or male factor and no ovarian factor), NOF (women between 27 and 39 yo with tubal or male factor and no ovarian factor), EM (endometriosis), PR (women < 40 yo defined as poor responders) and PCOS (polycystic ovarian syndrome).

**Table 2:** Expression levels of sirtuin-1–7 genes by diagnostic group.

	Study 1			Study 2			
Gene	OD	NOF	≥40	NOF	EM	PR	PCOS
*SIRT1*	225.2 ± 46.0 ^a^	169.1 ± 11.7 ^a^	478.3 ± 111.1 ^b^	169.1 ± 11.7 ^a^	139.3 ± 16.2 ^a^	166.4 ± 30.2 ^a^	138.5 ± 36.4 ^a^
*SIRT2*	271.4 ± 36.6 ^a^	161 ± 16.1 ^a^	600.1 ± 97.0 ^b^	161 ± 16.1 ^a^	637.5 ± 57.1 ^b/c^	793.0 ± 97.7 ^b^	460.4 ± 68.5 ^c^
*SIRT3*	70.8 ± 8.9 ^a^	72.1 ± 3.1 ^a^	115.3 ± 27.6 ^a^	72.1 ± 3.1 ^a^	117.7 ± 14.7 ^a/b^	150.9 ± 34.3 ^b^	84.3 ± 14.3 ^a/b^
*SIRT4*	2.5 ± 0.6 ^a^	4.3 ± 0.3 ^a^	2.7 ± 0.9 ^a^	4.3 ± 0.3 ^a^	1.9 ± 0.2 ^a^	3.6 ± 0.9 ^a^	2.01 ± 0.6 ^a^
*SIRT5*	83.2 ±10.5 ^a^	79.2 ± 4.9 ^a^	171.6 ± 37.2 ^b^	79.2 ± 4.9 ^a^	143.8 ± 18.1 ^a/b^	169.7 ± 22.2 ^b^	146.8 ± 20.3 ^a/b^
*SIRT6*	3.2 ± 0.7 ^a^	4.4 ± 0.5 ^a^	2.5 ± 0.5 ^a^	4.4 ± 0.5 ^a^	6.3 ± 1.3 ^a^	13.8 ± 3.8 ^b^	4.7 ± 1.6 ^a^
*SIRT7*	32.7 ± 7.3 ^a^	68.6 ± 8.8 ^a^	66.5 ± 14.5 ^a^	68.6 ± 8.8 ^a^	104.9 ± 11.3 ^a^	151.2 ± 30.8 ^b^	91.4 ± 17.2 ^a/b^

Results were determined by qRT-PCR and are expressed as mean ± standard error. Different lowercase letters (a, b and c) represent statistically significant differences of the means in each study. Gene expression values are ×10^5^ relative to β-actin expression.

**Table 3:** RT-PCR primers for sirtuin genes (1–7) and β-actin.

Gene	Oligonucleotide	Sequence (5′→3′)	Tm (°C)
*SIRT1*	SIRT1-F	CTATACCCAGAACATAGACACG	54.1
	SIRT1-R	ACAAATCAGGCAAGATGC	54.5
*SIRT2*	SIRT2-F	CCATCTGTCACTACTTCATGC	55.8
	SIRT2-R	AAGTCCTCCTGTTCCAGC	55.1
*SIRT3*	SIRT3-F	GCTGGACAGAAGAGATGC	54.1
	SIRT3-R	GTGGATGTCTCCTATGTTACC	47.6
*SIRT4*	SIRT4-F	CAGATGTCGTTTTCTTCG	44.4
	SIRT4-R	CCAGAGTATACCTGCAAGG	52.6
*SIRT5*	SIRT5-F	CCCAGAACATCGATGAGC	55.6
	SIRT5-R	GCCACAACTCCACAAGAGG	57.9
*SIRT6*	SIRT6-F	AGGGACAAACTGGCAGAGC	60.4
	SIRT6-R	TTAGCCACGGTGCAGAGC	61.1
*SIRT7*	SIRT7-F	GCAGAGCAGACACCATCC	57.7
	SIRT7-R	GTTCACGATGTAAAGCTTCG	56.1
*β-Actin*	ACTB-F	CTTCCTTCCTGGGCATGG	61.6
	ACTB-R	GCCGCCAGACAGCACTGT	63.7

## Abbreviations

Days	Total number of days of stimulation
FF	Follicular fluid
FSH	Follicle stimulating hormone
hCG	Human chorionic gonadotropin
hGL	Human granulosa-lutein cells
hMG	Human menopausal gonadotropins
ICSI	Intracytoplasmic sperm injection
IVF	In vitro fertilization
LH	Luteinizing hormone
OD	Oocyte donors
NOF	Women with no ovarian factor
PCOS	Polycystic ovarian syndrome
Peak E2	Estradiol concentration on the last day of stimulation
PR	Poor responders
rFSH	Recombinant follicle stimulating hormone
rLH	Recombinant luteinizing hormone
SIRT	Sirtuin
UI	International unit
≥40 yo	Women ≥40 years old with tubal or male factor and no ovarian factor
